# Ezetimibe: Clinical and Scientific Meaning of the IMPROVE-IT Study

**DOI:** 10.5935/abc.20160033

**Published:** 2016-03

**Authors:** Luis C. L. Correia

**Affiliations:** Escola Bahiana de Medicina e Saúde Pública; Hospital São Rafael, Salvador, BA - Brazil

**Keywords:** Hypolipidemic Agents, Ezetimibe, Cholesterol, Hydroxymethylglutaryl CoA- Redutases, Drug Combination

## Ezetimibe: Clinical and scientific meaning of the IMPROVE-IT study

After years of anxious wait, in June of the current year, the results of the clinical trial IMPROVE-IT were published in the New England Journal of Medicine.^1^ This was the first study to properly evaluate the clinical impact of adding ezetimibe to statin therapy. Until then, we had known that the combination of ezetimibe with statin enhanced the antihyperlipidemic effect of statins (surrogate endpoint). However, a positive surrogate marker does not necessarily have a guaranteed clinical impact on the therapy.^2^ Therefore, the IMPROVE-IT would fill a gap in the literature concerning the effect of ezetimibe on clinical outcomes.

The publication of the ENHANCE study in 2008 created uncertainty about the efficacy of the therapy with ezetimibe.^3^ In this randomized, clinical trial including patients with familial hypercholesterolemia, the therapy with ezetimibe added to 80 mg of simvastatin led to greater reductions in LDL cholesterol levels as compared with simvastatin alone. However, the effect was not accompanied by a greater reduction in atherosclerosis, measured by the carotid intima-media thickness (surrogate endpoint). At this point, it became evident that the industry was precipitate in promoting the use of a drug before its effects were demonstrated by clinical outcomes.

In the same year, the New England Journal of Medicine published an article showing how the marketing to physicians and consumers had a positive impact on sales of ezetimibe in the American market, despite the absence of clinical evidence, differently from what was observed in Canada.^4^ The marketing was primarily aimed at substituting conventional therapeutic regimens with the combination of ezetimibe with statin at low doses. Nevertheless, in the lack of large studies to evaluate the impact on clinical outcomes, whether the substitution of low doses for high doses of statins (combined with ezetimibe) would lead to decreases in the pleiotropic effects of statins remained unknown.

Thus, the results of the IMPROVE-IT study were awaited. They were first presented at the American Heart Association's scientific sessions in November, 2014. In contrast to what usually occurs to large clinical trials, the study was not published on the day of presentation, which raised speculations that disagreements between authors and editors caused such delay in the publication, although the authors stressed that they had not submitted the study to the journal before the presentation at the meeting.

In this context, we will evaluate the dual meaning of this study, which demonstrated not only the efficacy of ezetimibe, but also only a slight decrease of the risk. This observation encourages us to have an intense debate over the relative risk reduction, the absolute risk reduction (ARR) and the number needed to treat (NNT) in the critical analysis of the scientific evidence.

## The Proof of Concept

The IMPROVE-IT study demonstrated that 10 mg of ezetimibe plus 40 mg of simvastatin reduced the incidence of clinical outcomes as compared with 40 mg of simvastatin in patients who had been recently suffered an acute coronary syndrome and had a low LDL cholesterol level (<125 mg/dL).

The methodology of the study has a high degree of confidence: low risk of bias, since it was a large study involving 18,000 randomized patients (homogeneity of the groups prevents confounding effects); double-blind (preventing performance bias or measurement bias due to biased observation); intention-to-treat analysis; and low loss to follow-up. With respect to the risk of random error, the study had adequate statistical power, based on the analysis of primary outcome (and not of a secondary outcome), on the analysis of the entire sample (and not of subgroups), and on the absence of early stopping of the trial for benefit (truncation).

Therefore, this study was the first demonstration of clinical protection with ezetimibe, by showing the efficacy of its addition to the therapy with statin therapy in increasing the antihyperlipidemic effect.

## The size of the effect

At the same time that the IMPROVE-IT study demonstrated the efficacy of ezetimibe, it also showed that such effect was almost irrelevant. In fact, this was a positive study concerning the presence of the effect, but negative in relation to the size of the effect.

My point of view may sound strange, since the NNT of the IMPROVE-IT was 50, which is considered of moderate relevance. For example, with this NNT found in the CURE study,^5^ we started to combine clopidogrel with acetylsalicylic acid in patients with acute coronary syndromes. So why am I saying that the IMPROVE-IT is a negative study regarding the size of the effect? The answer is based on the relative risk reduction of only 6%.

The (correct) notion that has been supported by the evidence-based medicine is that the relative risk reduction alone may lead to an overestimation of the benefits of certain treatment, which makes us to calculate the ARR and the NNT (100/ARR) for a better overview of the effect. Indeed, authors have more commonly used relative risk reductions than absolute reductions,^6^ since beneficial treatments have relative reductions of 20%-40% whereas absolute reductions of approximately 2%-4%, which makes the former more attractive to use.

The lower value of the ARR not only makes it a more reliable parameter, but also approximates it to the real impact. For example, the information that I have received 50% of an inheritance (relative value) has little meaning if I do not know the total value of the inheritance (absolute value). However, when the relative risk reduction is low, both the ARR and the NTT may overestimate the benefits of the treatment. The roles are reversed. Why?

The relative risk reduction represents an intrinsic effect of the therapy, and tends to be constant, independently of the patient's baseline risk.^7^ In contrast, the ARR is not an intrinsic property, and varies accordingly with the baseline risk. For the same relative reduction, the higher the baseline risk, the higher the absolute risk. Therefore, when the intrinsic effect of the therapy is minimal, the authors may exacerbate the results and make them seem relevant by applying them in a sample in which the incidence of the outcome is high.

That was what occurred in the IMPROVE-IT study, which compared a good treatment (statin) with a mere improvement of such treatment (combination with ezetimibe) in patients with low cholesterol (<125 mg/dL). The expected intrinsic effect could be nothing but minimal. Once aware of this, the authors have chosen patients with acute coronary syndromes (higher risk) and defined outcome as the combination of five components (death, infarction, stroke, hospitalization for angina and need of revascularization), in place of the three traditionally used components (death, infarction and stroke). Consequently, the incidence of the outcome was 34%, and a slight relative reduction of 6% would lead to a reasonable absolute reduction of 2% and a NNT of 50.

It is usually argued that ARR and NNT are more representative parameters of relevance, since it involves both the relative and the absolute reduction (relative risk reduction x absolute risk = ARR). However, when a very low relative reduction is accompanied by a reasonable NNT, we need to be cautious. There are two possibilities:

First, the NNT is artificially exacerbated by the study sample, composed by a subgroup of patients with high risk, or by an outcome composed of many items, some of them less relevant than others. Both of these occurred in the IMPROVE-IT study - a population with high incidence of the outcome (acute coronary syndromes) had been chosen in order to clinically validate the use of ezetimibe; and second, the outcome was a combination of death, infarction, stroke (hard outcomes), hospitalization for angina and need of revascularization (soft outcomes). Of the 170 outcomes prevented, only one death was prevented, and the others included infarction and hospitalization.

In the second possibility, the NNT correctly reflects the magnitude of the effect, because the risk of the disease is too high, and even a small relative risk reduction has a relatively good impact. This is what happens with the angiotensin-converting-enzyme (ACE) inhibitor in heart failure, which promotes 16% of relative risk reduction.^8^ However, this is a high-mortality disease, leading to a high NNT. Here, there is no selection of a specific subgroup or a combination of many outcomes. We are only speaking of death.

In light of this, we can follow the following rule: if the relative risk reduction is reasonable (>20%), we should calculate the NNT, which will indicate whether the intrinsic effect reflects a relevant absolute difference. Or, if the relative risk reduction is low, we may conclude that the impact to the treatment is also low, and this should be reserved to situations of high incidence of the outcome. Thus, the analysis of the size of the effect should take into account both the relative risk reduction (intrinsic effect) and the impact on the mean patient (NNT).

## Imprecision of the size of the effect

Another aspect of the IMPROVE-IT is the imprecision of its estimates. We are mentioning not only a relative risk reduction of only 6%, but also a confidence interval of 1%-11%. In other words, we cannot rule out the possibility of a relative risk reduction as low as 1%. Considering this worse scenario, the NNT would be of 288. Although the IMPROVE-IT is a large study (n=18,000), it becomes inaccurate in describing such a small effect.

## Clinical implications

The IMPROVE-IT study demonstrated that the prescription of ezetimibe for patients in use of statins and reasonable cholesterol levels (mean LDL = 90 mg/dL) has a minimal beneficial effect. Then, the prescription of this combination would be an exception, rather than a rule, limited to unusual situations in which a relative risk reduction of 6% would promote an absolute risk reduction of satisfactory magnitude of hard outcomes. The minimal effect presented by the study implies that the treatment may be used in a minority of patients. For this reason, we believe that the IMPROVE-IT study yielded a more negative than positive result.

Considering the evidence-based medicine paradigm, the utilization of indirect evidence can be considered adequate. And this was the main contribution of the IMPROVE-IT, i.e., the application of the concept proved by the study in other types of patients, including, for example, those who remain with high cholesterol levels despite statin therapy at maximum doses. This was not the focus of the IMPROVE-IT, although its results indirectly indicate that the combination with ezetimibe may reduce the occurrence of events in these patients - if the effect was observed in patients with normal cholesterol levels, why it would not be expected in patients with high cholesterol levels? According to the meta-analysis graph depicted in the discussion session of the IMPROVE-IT, the higher the ARR of cholesterol level, the higher the relative risk reduction.

Therefore, if we use the concept of the IMPROVE-IT in a population with higher cholesterol level, we may achieve a higher benefit; that would be a good example of using indirect evidence. This tends to be a more useful application, since we would be correcting a still elevated cholesterol level despite the use of statin. This is the case in which the use of indirect evidence seems to make more sense than the use of direct evidence.

In addition, in the IMPROVE-IT study, ezetimibe was combined with a statin therapy of moderate potential (40 mg of simvastatin). Wouldn't it be better to substitute it with a more potent regimen, using high doses of statin, as was performed in the PROVE-IT study?^9^ We do not have this answer. In case a more potent statin regimen was already being used, then the therapy could be enhanced by ezetimibe. This is another argument in favor of the indirect use of evidence, differently from what was described in the IMPROVE-IT study. However, this also seems to be an exception indication.

Therefore, for the above-mentioned reasons, the clinical applicability of the IMPROVE-IT study is limited.

## The Real Value of the IMPROVE-IT

The greatest value of the IMPROVE-IT study is its scientific message that corroborates the hypothesis that cholesterol is a cardiovascular risk factor. Statins reduce cholesterol levels and the cardiovascular risk, representing a criterion of reversibility and supporting this hypothesis. However, some critics argue that statins reduce cardiovascular risk by their pleiotropic effects rather than by reducing cholesterol levels. The IMPROVE-IT demonstrated that the additional reduction in cholesterol levels by a drug other than statin also reduce the cardiovascular risk, hence rejecting the null hypothesis of the relationship between cholesterol and cardiovascular risk.

And, sorry for the pun, but what the IMPROVE-IT actually improves is our scientific maturity.
